# Incident diabetes, hypertension and dyslipidemia in a Manitoba First Nation

**DOI:** 10.3402/ijch.v74.27712

**Published:** 2015-08-20

**Authors:** Natalie D. Riediger, Virginia Lukianchuk, Sharon G. Bruce

**Affiliations:** 1 Department of Community Health Sciences, University of Manitoba, Winnipeg, MB, Canada; 2 Manitoba First Nations Centre for Aboriginal Health Research, University of Manitoba, Winnipeg, MB, Canada; 3 Sandy Bay Health Centre, Sandy Bay Ojibway First Nation, Marius, MB, Canada

**Keywords:** First Nations, diabetes, incidence, community-based participatory research

## Abstract

**Background:**

Diabetes and diabetes complications are substantially higher among Canadian First Nations populations compared with the general Canadian population. However, incidence data using detailed individual assessments from a population-based cohort have not been undertaken.

**Objective:**

We sought to describe incident diabetes, hypertension and dyslipidemia in a population-based cohort from a Manitoba Ojibway First Nation community.

**Design:**

Study data were from 2 diabetes screening studies in Sandy Bay First Nation in Manitoba, Canada, collected in 2002/2003 and 2011/2012. The cohort comprised of respondents to both screening studies (n=171). Health and demographic data were collected using a questionnaire. Fasting blood samples, blood pressure and anthropometric data were also collected objectively. Incident diabetes, hypertension and dyslipidemia were determined. Generalized linear models with Poisson distribution were used to estimate risk of incident diabetes and cardiometabolic conditions according to age and sex.

**Results:**

There were 35 (95% CI: 26, 45) new cases of diabetes among 128 participants without diabetes at baseline (27 or 3.3% per year). While participants who were 50 years and older at baseline had a significantly higher risk of incident diabetes at follow-up compared with participants aged 18–29 at baseline (p=0.012), more than half of the incident cases of diabetes occurred among participants aged less than 40 at baseline. There were 28 (95% CI: 20, 37) new cases of dyslipidemia at follow-up among 112 without dyslipidemia at baseline (25%). There were 36 (95% CI: 31, 42) new cases of hypertension among 104 participants without hypertension at baseline (34.6%). Women had half the risk of developing hypertension compared with men (p=0.039).

**Conclusions:**

Diabetes incidence is very high, and the number of new cases among those younger than 40 is a concern. Additional public health and primary care efforts are needed to address the diabetes burden in this First Nation community.

Prevalence of type 2 diabetes is increasing worldwide for various reasons (1). However, indigenous populations have been more adversely affected (2–5). In Canada, many First Nations people live in environments that promote development of chronic diseases, such as diabetes and heart disease. Many aspects of this environment are rooted in the legacy of colonialism and include limited incomes (6), low educational attainment (7), limited access to affordable foods (8), reduced access to health care (9) and racism (2).

In Manitoba, the prevalence of diabetes is over 4 times higher among First Nations people compared with non-First Nations people (3). Similar gaps in prevalence of diabetes have been noted more recently among First Nations populations compared with non-First Nations people in Saskatchewan (4) and Alberta (5), with higher prevalence noted among First Nations women compared with men (4, 5). Other studies based on the First Nations community have also reported high prevalence of diabetes (10–12). In the study community of Sandy Bay First Nation, Manitoba, the diabetes burden is similarly heavy, with an age- and sex-standardized prevalence of 39% among adults (7). Furthermore, the study community is within the Dakota Ojibway Tribal Council, which has the highest adjusted prevalence of diabetes among adults compared with all other Tribal Councils in Manitoba, at approximately 25% (9).

While diabetes prevalence has been well described in Canadian First Nations populations (3–5, 7, 10–12), incidence has not, particularly recent estimates. Incidence is important to understanding the trajectory of the epidemic (i.e. Are people continuing to develop diabetes and, if so, how is incidence distributed within the population?). Among researchers who have reported on incidence, estimates were derived from either administrative data (3, 4) or hospital registries (10). Chart review, administrative and registry data have many strengths but one disadvantage of each is that they rely on diagnosed cases, and the issue of undiagnosed cases, particularly in marginalized populations, is well established (13, 14). To our knowledge, there has been only one Canadian longitudinal study with First Nations people (Sandy Lake, ON) that included measures of glucose metabolism at baseline and follow-up; however, the sample size was small (n=95), follow-up was 4 years and the sample was not based on population but consisted of high-risk individuals only (15, 16). Population-based studies are required to determine incidence of diabetes, particularly in communities where diabetes burden is high and the age of onset is young. In addition, previous studies from Sandy Lake First Nation that reported diabetes incidence did not report incident hypertension or dyslipidemia (17–19), yet these cardiometabolic conditions are important contributors to the development of diabetes, diabetes complications and other chronic disease, particularly in the First Nations population. Therefore, the purpose of the study was to determine incidence rates of diabetes, hypertension and dyslipidemia in a population-based cohort from one Manitoba First Nation community.

## Methods

### Framework

This study was approached using a community-based participatory research (CBPR) framework. Briefly, we have applied the core aspects of CBPR described by Israel et al. (20): (a) the participatory component is to take into account local context; (b) there should be co-operation and equal contribution between community members and researcher(s); (c) co-learning of both the researcher(s) and community; (d) capacity building in the local community and expanding the “strength and resources in community,” which included 4 community research assistants; (e) empowerment to reduce social inequities; (f) a balance between research and action or policy change, which may or may not include a direct “action” component, so that all partners can benefit. Initially, the community identified the health issues and sought out university researchers; a Community Diabetes Advisory Group was then established. The Community Diabetes Advisory Group includes members of the community health centre, community members and university researchers. In this regard, the community, by way of a Community Diabetes Advisory Group, was involved in the design, data collection, analysis, interpretation, presentation and publication of the study results. The group met regularly prior to, during, and following data collection to discuss and plan all these components.

### Design

The study community is Sandy Bay Ojibway First Nation, located in southwest Manitoba, approximately 200 km northwest of Winnipeg. This community is accessible year round by road. The total on-reserve population in 2011 was approximately 4,100 people with 50% younger than 19 years.

All adults, 18 years and older and non-pregnant, were invited to a Diabetes Screening Study in 2002/2003 (convenience sample) (7); 36% of the eligible population participated (n=1,346) (7). Inclusion criteria included being a registered member of Sandy Bay First Nation or a registered member of another First Nation but living in Sandy Bay. Eligible individuals were invited through advertisements on the community radio station and health centre, as well as home visits from community research assistants. Two of the three community research assistants were elders and familiar with the majority of the residents in the community. This sample was representative of the community population in 2002/2003 according to age, sex and employment (7). In 2011/2012, a second Diabetes Screening Study was completed (13). Targeted recruitment of participants from the 2002/2003 sample was employed to optimize the longitudinal sample size. Specifically, we identified participants from the 2002/2003 study who were known to be alive and residing in the community; community research assistants then personally invited those participants to return for the 2011/2012 study through home visits. The community research assistants who were also elders were able to identify those that had passed away or moved. The studies were approved by the University of Manitoba Health Research Ethics Board (H2001:178; H2011:171).

### Data collection and laboratory procedures

In both 2002/2003 and 2011/2012, venous blood samples were drawn by a registered nurse after a minimum 12-h fast, processed on site at the health centre and stored at −20°C. Details of the biochemical analysis have been previously described (7, 13). Briefly, fasting glucose levels were determined using the hexokinase/glucose-6-phosphate dehydrogenase assay. Lipids were determined using enzymatic colorimetric methods. Two blood pressure readings were taken after a 5 min rest, both initially and between readings, and averaged. All anthropometric measures were taken by trained research assistants using established methods previously described (7, 13). Participants’ medical histories, including previous diagnosis of diabetes or hypertension, as well as medication use, were obtained by questionnaire administered by a research assistant.

### Definition of cardiometabolic conditions

Diabetes is defined as currently on an oral hypoglycaemic, self-declared or fasting glucose ≥7.0 mmol/L (21, 22). Impaired fasting glucose (IFG) is defined as a fasting glucose between 6.1 and 6.9 mmol/L (21). Hypertension is defined as a previous diagnosis of hypertension; or systolic blood pressure (SBP) >140 mm Hg or diastolic blood pressure (DBP) >90 mm Hg; or for participants with diabetes, SBP≥130 mm Hg or DBP≥80 mm Hg (21, 22). Dyslipidemia is defined as a fasting plasma triglycerides ≥1.7 mmol/L, in combination with a fasting plasma high-density lipoprotein cholesterol (HDL-C) <1.03 mmol/L (for men) or plasma HDL-C<1.3 mmol/L (for women) (13, 23).

### Statistical analyses

First, descriptive analysis of the 2002/2003 sample and the longitudinal cohort at baseline were completed according to age group, sex, highest level of education complete, employment status, diabetes, obesity, hypertension and dyslipidemia. Age groups were: 18–29, 30–39, 40–49 and 50 years and older. Highest level of education completed was dichotomized according to median grade level in the 2002/2003 sample. Employment status included any work for pay, including part-time and full-time. Obesity is defined as body mass index ≥30 kg/m^2^. Differences between the baseline sample and the sample with follow-up data were tested using chi-square statistic and independent sample *t*-test. We acknowledge that the 2 samples are not independent; however, mixed models accounting for repeated measurements did not converge.

The number of new cases of each of the relevant conditions was determined together with a 95% CI to demonstrate the reliability of the estimate for the population. Only participants without the respective condition at baseline were included in the denominator. The number of incident cases of diabetes was also identified as either previously diagnosed during the follow-up period or detected at screening (i.e. undiagnosed). Incidence density was also determined, assuming a diagnosis at mid-point of follow-up; this assumes incidence was constant throughout the study period. We acknowledge that incidence density, in this case, has several limitations; it is reported to facilitate comparison to the literature.

Age group, age as a continuous variable, and sex were explored as potential predictors of incident disease (diabetes, hypertension and dyslipidemia) using a generalized linear model with Poisson distribution. The relative risk according to age group and sex is reported, not controlling for the other in each respective analysis. All statistical analyses were conducted using the current version of SPSS (version 22) with significance set at p<0.05.

## Results

Of 478 participants from 2002/2003, 171 returned in 2011/2012 comprising 36% of the original sample. Similarly, 37% of participants without diabetes at baseline returned at follow-up (n=128). Although attempts were made to recruit the 2002/2003 participants for the 2011/2012 study, a larger number than anticipated moved, passed away, refused to participate or could not be located. A description of the study sample at baseline is provided in Table I. In addition, a comparison to the overall sample in 2002/2003 was included to verify the representativeness of the cohort. The prospective cohort was 2 years younger compared with the baseline sample, which was mostly accounted for by fewer participants aged older than 50 years at baseline. However, the 2 samples were not significantly different according to sex, education, employment, marital status, diabetes, obesity or hypertension prevalence at baseline.

**Table I:** Description of longitudinal study sample at baseline compared with the 2002/2003 sample

Variables	Longitudinal sample (n=171)	2002/2003 sample (n=478)	p
Age	35.7±9.6	37.8±12.3	0.041^a^
Age groups at enrolment			0.009
18–29 years	48 (28.1)	142 (29.5)	
30–39 years	65 (38.0)	144 (29.9)	
40–49 years	44 (25.7)	108 (22.4)	
≥50 years	14 (8.2)	88 (18.3)	
Sex			0.834
Men	80 (46.8)	230 (47.7)	
Women	91 (53.2)	252 (52.3)	
Highest level of education^b^			0.465
<grade 9	83 (49.7)	248 (53.0)	
≥grade 9	84 (50.3)	220 (47.0)	
Employed			0.237
Yes	41 (24.1)	137 (28.8)	
No	129 (75.9)	338 (71.2)	
Diabetes			0.329
Yes	43 (25.1)	140 (29.0)	
No	128 (74.9)	342 (71.0)	
Hypertension			0.510
Yes	64 (37.9)	192 (40.8)	
No	105 (62.1)	279 (59.2)	
Obese			0.940
Yes	94 (56.3)	265 (56.6)	
No	73 (43.7)	203 (43.4)	

Data are presented as either mean (SD) or *n* (%). The p-value refers to the result from a chi-square test unless otherwise noted.

a The p-value refers to the result from an independent sample *t*-test.

b Highest level of education was dichotomized according to median in 2002/2003.

### Diabetes

There were 35 (95% CI: 26, 45) new cases of diabetes among 128 participants without diabetes at baseline (27 or 3.3% per year); the incidence density is 38.6 cases/1,000 person-years. Thirty-four per cent (12/35) of the incident cases were newly identified at screening, that is, undiagnosed. Among the 13 cases of IFG at baseline, 9 cases had progressed to diabetes (69 or 8.4% per year). Among participants with normal glucose tolerance at baseline (n=115), 10 (95% CI: 5, 16) had progressed to IFG and 26 (95% CI: 18, 35) to diabetes.

Compared with participants aged 18–29 at baseline, participants aged 50 years and older had 4 times the risk of developing diabetes (p=0.012) (Table II). Participants 40–49 years old had more than 2 times the risk of developing diabetes compared with the youngest age group; however, this trend did not reach significance (p=0.078). Another important note regarding the age of incidence cases was that more than 50% of new diagnoses were among participants less than 40 years old at baseline. Risk of incident diabetes was not significantly different between men and women (p=0.771).

**Table II:** Risk of incident diabetes, hypertension and dyslipidemia according age and sex

Condition	Characteristic	Incident cases	Number at risk	Relative risk (95% CI)	p
Diabetes	Age at enrolment				
	18–29 years	9	44	Reference	
	30–39 years	9	51	0.863 (0.342, 2.173)	0.754
	40–49 years	12	27	2.173 (0.916, 5.157)	0.078
	≥50 years	5	6	4.074 (1.365, 12.156)	0.012
	Age (continuous)			1.051 (1.013, 1.091)	0.008
	Sex				
	Men	15	58	Reference	
	Women	20	70	1.105 (0.566, 2.158)	0.771
Hypertension	Age				
	18–29 years	13	41	Reference	
	30–39 years	12	42	0.901 (0.411, 1.975)	0.795
	40–49 years	8	18	1.402 (0.581, 3.382)	0.452
	≥50 years	3	3	3.154 (0.899, 11.067)	0.073
	Age (continuous)			1.033 (0.994, 1.073)	0.101
	Sex				
	Men	24	51	Reference	
	Women	12	53	0.481 (0.241, 0.962)	0.039
Dyslipidemia	Age				
	18–29 years	13	38	Reference	
	30–39 years	5	41	0.356 (0.127, 1.000)	0.050
	40–49 years	7	25	0.818 (0.327, 2.051)	0.669
	≥50 years	3	8	1.096 (0.312, 3.847)	0.886
	Age (continuous)			1.000 (0.963, 1.039)	0.985
	Sex				
	Men	12	59	Reference	
	Women	16	53	1.484 (0.702, 3.137)	0.301

### Hypertension

There were 36 (95% CI: 31, 42) new cases of hypertension among 104 participants without hypertension at baseline (34.6%). Age was not significantly associated with incident hypertension as either a categorical or continuous variable (Table II). Also, women had half the risk of developing hypertension compared with men (p=0.039).

### Dyslipidemia

There were 28 (95% CI: 20, 37) new cases of dyslipidemia at follow-up among 112 participants without dyslipidemia at baseline (25%). Risk of dyslipidemia was not significantly different between the age groups or sex (Table II). Of note, 46% (13/28) of new cases of dyslipidemia were among participants 18–29 years old at baseline.

## Discussion

In the present study, the cumulative incidence of diabetes was 27% over 8.2 years and the incidence density was 38.6 cases/1,000 person-years. The higher rate among participants 50 years and older reported here is consistent with the previous report in Manitoba First Nations in which diabetes risk increases with age (3). Incidence density of diabetes reported among other indigenous populations (3–5, 24–26) is summarized in Table III. Incidence rates have varied across different indigenous groups and over time. In Sandy Lake First Nation, an incidence rate of 17.5% over 10 years among individuals ≥10 years old at baseline was reported between 1995 and 2005 (17). The incidence rate in the study community confirms a high rate in another First Nation group, as well as the persistence of a high incidence in a later time period as well as perhaps an increase in incidence over time. While the incidence rate appears to be highest in the study community, direct comparisons between studies are not possible due to different dates surveyed, age groups included, age structure of the population and/or method of diabetes identification.

**Table III:** Comparison of diabetes incidence rates in Indigenous populations

Population	Incidence rate per 1,000 person-years	Age at enrolment	Time period	Method of diabetes identification	References
Sandy Bay First Nation (Manitoba, Canada)	38.6	≥18 years old	2002/2003–2011/2012	Self-report and fasting glucose	Present study
Australian Aboriginal communities (remote, central)	20.3	15–77 years old	1987–1995	2-h oral glucose tolerance test	(24)
Manitoba First Nations population		≥20 years old	1994–1998	Administrative data	(3)
Men	21.1				
Women	21.1 (estimated based on other data provided)				
Albertan First Nations population		≥20 years old	1995–2007	Administrative data	(25)
Men	10.3				
Women	11.9				
Saskatchewan First Nations population		≥20 years old	1980–2005 (incidence density	Administrative data	(4)
Men	17.80		for 2003 reported)		
Women	17.95				
Kahnawá:ke First Nation		≥18 years old	1986–1988	Administrative data	(10)
Men	8.8		2001–2003		
Women	8.8				
Men	7.0				
Women	5.2				
American Pima Indians			1991–2003	2-h oral glucose	(26)
(Gila River Indian	9.4	15–24 years old		tolerance test	
Community in Arizona)	22.6	25–34 years old			
	43.4	35–44 years old			
	49.8	45–54 years old			
	70.8	55–64 years old			
	43.4	≥65 years old			

Incident diabetes was not significantly different between men and women in the present study. In contrast, Green et al. (3) reported a higher diabetes incidence among First Nations women compared with First Nations men in Manitoba. However, the authors also reported an increasing incidence of diabetes over time for First Nations men compared with a plateau for First Nations women in Manitoba during the 1990s; this finding supports the reduction in the diabetes gap between First Nations men and women over time and as the epidemic advances. We have also reported no sex difference in diabetes prevalence in the study community in either 2002/2003 or 2011/2012 (13).

An important finding with regard to diabetes and all conditions is the high burden of risk among young adults. Over 50% of incident cases of diabetes were among participants less than 40 years old at baseline. Like diabetes, we would expect incident dyslipidemia and hypertension to increase with age; however, this was not the case. These findings are consistent with previously published results from the cross-sectional portion of these studies, which highlight the burden of disease among young adults (13, 27–29). Due to the longitudinal design of this study, this finding supports the persistence of developing disease among young adults in this population. The burden of risk among young adults is also important with respect to diabetes complications; younger age of onset of type 2 diabetes is associated with greater risk of diabetes complications (30) and a higher risk of mortality and complications compared with type 1 diabetes (31). In addition, undiagnosed diabetes continues to be a problem in the study community with a third of incident cases being previously undiagnosed. We have previously reported that in the 2002/2003 sample, 24% of those with diabetes were unaware of their condition (27). Diagnosis of diabetes is critical in managing blood glucose and preventing/delaying complications (14).

Men had a 50% higher risk of developing hypertension compared with women. There was a sex difference in prevalence of hypertension in 2011/2012 that was not detected in 2002/2003 (13). This sex difference in prevalence may be partly explained by the difference of incidence of hypertension between men and women reported here. It appears as though changes in blood pressure operate independently of adiposity and lipid metabolism in this population given the differing patterns (both prevalence and incidence) of hypertension and other cardiometabolic conditions (13). It is also possible that the changes in prevalence of hypertension may reflect changes in lifestyle habits in the population. In this regard, the prevalence of smoking increased among men in the community, but not women (28). Another potential contributor is sex differences in health service use; men, in general, are less likely to seek primary care. In addition, family physician availability in the community is limited with physician services available one half-day per week (Joanne Roulette, Director of the Health Centre, 2011, oral communication, 16th of April). This is also a young population with a high fertility rate (Joanne Roulette, Director of the Health Centre, 2011, oral communication, 16th of April), which leads to greater screening for hypertension among women. On another note, the higher incidence of hypertension among men may partially explain the higher risk of end-stage renal disease observed among First Nation men compared with women despite higher rates of diabetes among First Nation women compared with men (32).

The results presented here are important for the Sandy Bay community health centre to govern their services. Given the study community is rural and has year-round access to urban centres, they operate under a health centre model. For this reason, the structure of services in place (i.e. a walk-in clinic type model) is not designed to address the needs of individuals who are chronically ill. Furthermore, with such a high burden of disease, the focus of the health centre is on treatment, which results in being in the difficult position of having to direct limited resources to what is supposed to be their prime mandate: prevention (Joanne Roulette, Director of the Health Centre, 2014, oral communication, 14th of April).

Strengths of this study include the CBPR framework and the richness of the data, including diabetes screening rather than use of administrative data. Limitations include the use of a convenience sample at baseline which may not be representative of the population based on factors other than age, sex or employment; the large loss-to-follow-up and subsequent small sample size. The large loss-to-follow-up may be partially attributed to the challenges of conducting studies in a rural community and may have resulted in underreporting of incident cardiometabolic conditions. Specifically, young adults from the community tend to be very geographically mobile (Joanne Roulette, Director of the Health Centre, 2012, oral communication, 5th of June). Limitations specific to estimating incidence density include the assumption of a mid-point time of diagnosis given that diabetes incidence is not consistent over time (33) and the limited measurement of exposure time for the majority of the population, given the large loss-to-follow-up. The use of fasting glucose as opposed to an oral glucose tolerance test is also considered a limitation, as incidence may be underestimated (34). Finally, the results of this study have limited external generalizability. However, it is unlikely that the results presented are unique to this First Nation community given similar risk factors and sociodemographic characteristics in some Manitoba communities and others in Canada.

In conclusion, the incident rate of diabetes, hypertension and dyslipidemia in this population are high compared with other First Nations populations, especially given the young age structure of the population. Although this is the first report of incidence in the community, these results indicate the persistence of disease among young adults previously reported (13, 29, 30, 34) and suggest that a high burden of diabetes complications in the study community will continue. These results are needed by the study community to prioritize their resources and importantly to advocate for a restructuring of their funding to accommodate the disease burden.
